# Co-localisation of the blackleg resistance genes *Rlm2* and *LepR3* on *Brassica napus* chromosome A10

**DOI:** 10.1186/s12870-014-0387-z

**Published:** 2014-12-31

**Authors:** Nicholas J Larkan, Derek J Lydiate, Fengqun Yu, S Roger Rimmer, M Hossein Borhan

**Affiliations:** Saskatoon Research Centre, Agriculture and Agri-Food Canada, 107 Science Place, Saskatoon, S7N 0X2 SK Canada

**Keywords:** Blackleg, *Brassica napus*, *Leptosphaeria maculans*, Marker-assisted breeding, Molecular marker, PGIP-like protein, Receptor-like protein, Resistance

## Abstract

**Background:**

The protection of canola (*Brassica napus*) crops against blackleg disease, caused by the fungal pathogen *Leptosphaeria maculans*, is largely mediated by race-specific resistance genes (*R*-genes). While many *R*-genes effective against blackleg disease have been identified in *Brassica* species, information of the precise genomic locations of the genes is limited.

**Results:**

In this study, the *Rlm2* gene for resistance to blackleg, located on chromosome A10 of the *B. napus* cultivar ‘Glacier’, was targeted for fine mapping. Molecular markers tightly linked to the gene were developed for use in mapping the resistance locus and defining the physical interval in *B. napus. Rlm2* was localised to a 5.8 cM interval corresponding to approximately 873 kb of the *B. napus* chromosome A10.

**Conclusion:**

The recently-cloned *B. napus R*-gene, *LepR3,* occupies the same region of A10 as *Rlm2* and analysis of the putative *B. napus* and *B. rapa* genes in the homologous region identified several additional candidate defense-related genes that may control *Rlm2* function.

## Background

When plants are under attack from fungal pathogens, they can often detect the secretion of fungal effector proteins, either directly or indirectly, by means of plant resistance (*R*) genes, which initiate a defense response known as effector-triggered immunity (ETI). ETI often causes localized cell death described as a hypersensitive response (HR) and prevents further infection [1,2]. *R*-genes are of vital importance in providing protection from plant pathogens, and an understanding of the relationship between race-specific *R*-genes and their corresponding pathogen avirulence (*Avr*) genes [3] is required for the effective deployment of resistance genetics in crop varieties. The hemibiotrophic fungal pathogen *Leptosphaeria maculans* (Desmaz.) Ces. & De Not. (anamorph: *Phomalingam* (Tode ex Fr.) Desmaz.) is the causal agent of blackleg disease; the most economically important disease of *Brassica* crops worldwide [4]. Eighteen major *R*-genes for blackleg disease have been identified in *Brassica* species, though several of these are probably redundant (reviewed in [5]). Most of the *R*-genes effective against blackleg described to date map to one of two chromosomes in the *Brassica* A genome; *Rlm1*, *Rlm3*, *Rlm4*, *Rlm7* and *Rlm9* form a cluster of *R*-genes on chromosome A07 [6-8], while *LepR2* [9], *BLMR2*/*RlmS* ([10], Larkan et al. unpublished]) *LepR3* [11] and *Rlm2* [6] map to chromosome A10. *Rlm2* was identified from the European *Brassica napus* (canola/rapeseed) variety ‘Glacier’ [12], which was later shown to contain two *R*-genes; *Rlm2* and *Rlm3* [13]. *LepR3* was identified from the *B. napus* cultivar “Surpass 400” [14] and was reportedly introgressed into *B. napus* from *B. rapa* subsp. *sylvestris* [15,16]. *LepR3* is to date the only *R*-gene for resistance to blackleg to be cloned. It encodes a receptor-like protein (RLP) which conveys resistance via HR during infection by *L. maculans* isolates expressing the *AvrLm1* avirulence gene [11]. The gene ‘BnaA10g20720D’ on chromosome A10 of the newly-sequenced *B. napus* reference ‘Darmor-*bzh*’ genome [17], homologous to the *B. rapa* gene Bra008930 [18], shares 99% identity with the susceptible *lepR3* allele of ‘Topas DH16516’ [11].

*Rlm2* confers race-specific resistance to *L. maculans* isolates harbouring the corresponding avirulence gene *AvrLm2* [12], which forms part of a genetic cluster of avirulence genes (*AvrLm1-2-6*) within the *L. maculans* genome [13]. Previously, the map location of *Rlm2* was investigated using two resistant cultivars; ‘Glacier’ and ‘Samouraï’, and shown to be positioned in the same interval of LG16 (corresponding to chromosome A10), confirming the same gene was present in both varieties. The gene was positioned within an interval of 12 cM on the ‘Darmor’ x ‘Samouraï’ LG16 (DS.16) map [6], though the lack of genomic resources available at the time prevented physical definition of the gene interval. *Rlm2* has been detected in many other European winter-type canola varieties, including one of the first blackleg-resistant cultivars ‘Ramsès’ [19], which was also used in the development of early Australian blackleg-resistant varieties [20]. *Rlm2* has also been detected in *B. rapa* [21].

The aim of the current study was to define the precise map location of *Rlm2*, and identify tightly-linked markers for use in marker-assisted breeding programs. By aligning the *B. napus* map interval containing *Rlm2* to the DNA sequence of *B. rapa* and *B. napus* for the corresponding region of chromosome A10 [17,18], we were able to physically define the interval containing the *Rlm2* locus on chromosome A10, determine the position of *Rlm2* relative to *LepR3* and identify several candidate defense-related gene homologues.

## Results

### Phenotypic analysis

**Figure 1:**
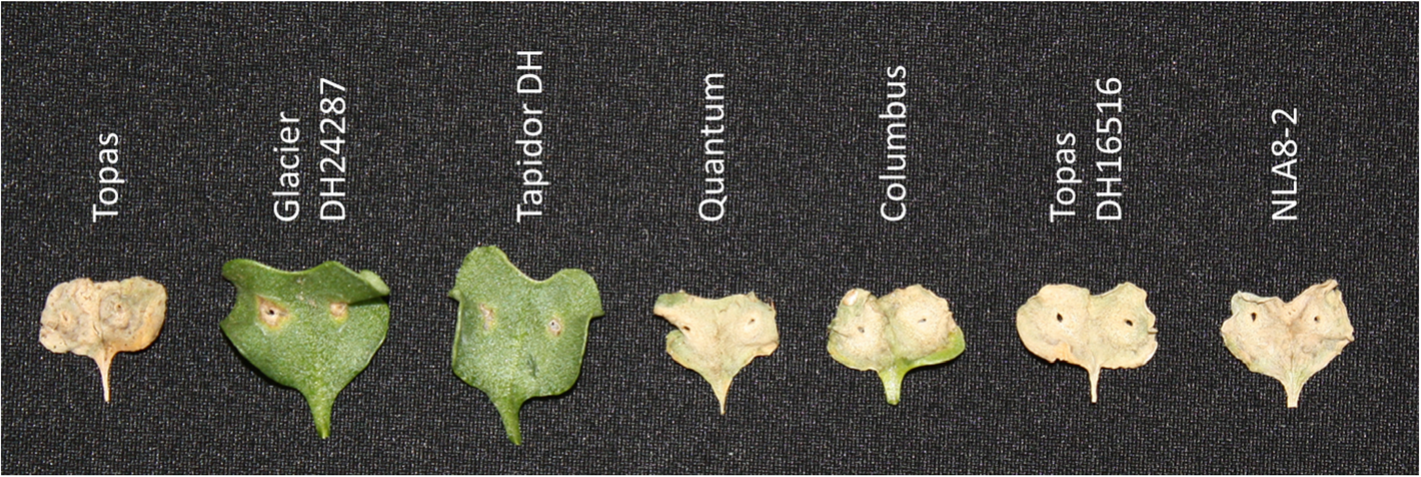
**Interaction of** ***L. maculans*** **isolate ‘165’ with differential lines of** ***B. napus*** **.** Cotyledons of seven *B. napus* lines pictured at 14-day post-inoculation. Lines containing *Rlm2* (‘Glacier DH24287’ – *Rlm2*, *Rlm3*; ‘Tapidor DH’ – *Rlm2*) showed typical hypersensitive response and restriction of lesions while lines absent *Rlm2* (‘Topas’ and ‘Topas DH16516’ – no blackleg resistance; ‘Quantum’- *Rlm3*; ‘Columbus’ – *Rlm1*, *Rlm3*; ‘NLA8-2’ – *LepR3*) were fully susceptible to infection.

The *L. maculans* isolate ‘165’ was used to inoculate 12 seedlings of each parental line used to construct the BC_1_F_1_ population. Infection with the isolate resulted in test scores between 7 and 9 on ‘Topas’ (average score 7.7) and scores of 2 to 5 on ‘Glacier DH24287’ (average score 2.5). Further tests using additional control *B. napus* lines showed ‘165’ was avirulent on the *Rlm2* line ‘Tapidor DH’ (2.1) and virulent on the *Rlm3* variety ‘Quantum’ (9.0), the *Rlm1*/*Rlm3* variety ‘Columbus’ (8.9), ‘Topas DH16516’ (9.0) and the Topas DH16516:*LepR3* transgenic line ‘NLA8-2’ (9.0). These results confirmed the “*avrLm1*, *AvrLm2*, *avrLm3*” pathotype of the isolate (Figure 1).

Phenotypic screening of the 940 BC_1_F_1_ (‘Topas’ x ‘Glacier’) individuals showed segregation for the *Rlm2* phenotype, with 478 seedlings scored as resistant to ‘165’ (scoring 2–5) and 462 seedlings scored as susceptible (7–9). This conformed to a 1:1 ratio (χ^2^ = 0.27, P = 0.602) as expected for a single dominant gene.

### *Rlm2* mapping

Twenty microsatellite markers spanning chromosome A10 were screened and a set of four markers (sR1448, sN8502, sN1982 & sN8474) that closely segregated with the *Rlm2* locus were selected for genotyping the Topas x Glacier DH24287 BC_1_F_1_ population. These markers spanned the equivalent of 240 genes (Bra008783 - Bra009023) and a physical interval of approximately 1 Mb of the *B. rapa* genome [18]. The selected microsatellite markers were used in conjunction with the initial 218 BC_1_F_1_ individuals to produce a draft map of the *Rlm2* interval using MapMaker v3.0b software [22] in order to confirm their linkage to the phenotype (LOD ≥ 4.0) and that they flanked the *Rlm2* locus. The remaining BC_1_F_1_ individuals were then screened for recombination within the *Rlm2* region. A total of 899 BC_1_F_1_ individuals where successfully genotyped with the flanking microsatellite markers.

Markers were re-run on the putative recombinant BC_1_F_1_ individuals and any non-confirmed recombinants discarded. After rescreening and phenotyping of the BC_1_F_2_ generation a total of 61 confirmed recombinants were retained for the informative recombinant mapping subset, each containing a single recombination event within the map interval.

**Figure 2:**
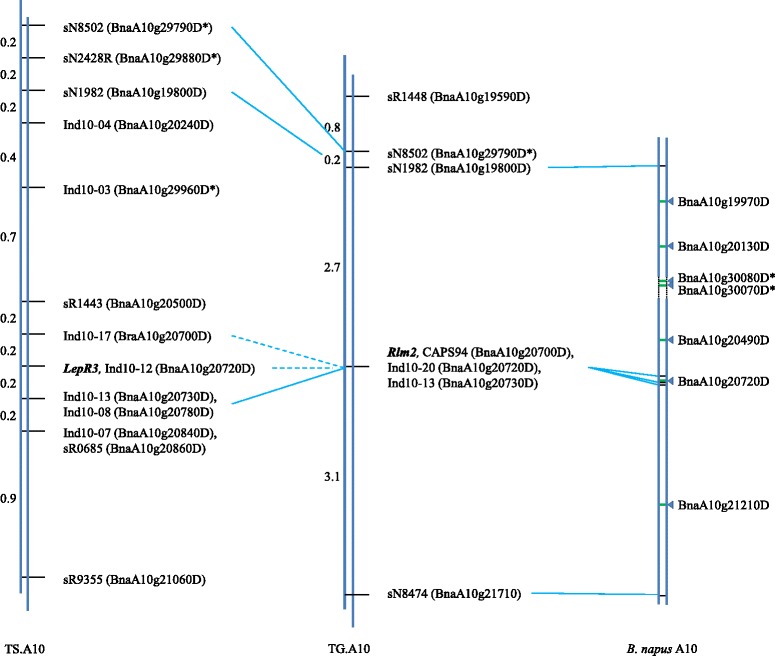
**Comparison of genetic maps for** ***Rlm2*** **and** ***LepR3*** **to** ***B. napus*** **chromosome A10.** Position of *Rlm2* relative to microsatellite (prefixed ‘sN’ or ‘sR’), Indel (prefixed ‘Ind’) and CAPS markers on ‘Topas’ x ‘Glacier’ A10 (TG.A10) map. Nearest *B. napus* gene to each marker given in brackets. Unanchored *B. napus* genes corresponding to syntenic *B. rapa* A10 homologues are denoted with “*”. *LepR3* ‘Topas’ x ‘Surpass 400’ A10 (TS.A10) map updated from Larkan et al., 2013a. Marker intervals given in centiMorgans. Solid horizontal lines denote same marker, dashed horizontal lines denote markers sharing same nearest *B. napus* homologue. Approximate physical location of defense-related candidate genes by triangles. Gap in *B. napus* A10 chromosome build represented by dotted lines.

The resulting map of ‘Topas’ x ‘Glacier’ A10 (TG.A10) showed *Rlm2* was contained within an interval of 5.8 cM, between sN1982 and sN8474 (Figure 2). This interval corresponded to a collinear span of approximately 926 kb of the *B. rapa* genome, containing 204 putative genes on chromosome A10 (Bra008819 to Bra009023). The majority of the cross-overs detected in the population (52 of 61) occurred either between sN1982 and the *Rlm2* locus (24 cross-overs), or between the *Rlm2* locus and sN8474 (28 crossovers). Two additional markers were designed for the *Rlm2* interval; one sequence characterised amplified region (SCAR) marker (Ind10-20), targeted to the Bra008930 homologue (previously identified as the *LepR3* locus) and one cleaved amplified polymorphic sequence (CAPS) marker (CAPS94), targeted to the Bra008928 homologue. Ind10-20 produced fragments of 142 bp for ‘Topas’ and 138 bp for ‘Glacier’. CAPS94 produces amplicons of approximately 900 bp from both parents. Digestion with BstUI produces fragments of 483 and 410 bp from the ‘Glacier’ A-genome amplicon only. When used to genotype the recombinant subset of the mapping population, both of these markers co-segregated with the *Rlm2* phenotype, providing markers tightly-linked to the *Rlm2* gene yet failing to reduce the size of the target map interval. An additional nine microsatellite markers positioned within the map interval were tested but also failed to provide additional informative data.

Comparison of the *Rlm2* and *LepR3* maps showed that both genes were located within the same genetic interval on chromosome A10. While one additional marker was integrated from the *LepR3* map (Ind10-13, corresponding to Bra008931), this also co-segregated with the *Rlm2* phenotype. All remaining markers used in the *LepR3* map were non-polymorphic in the ‘Topas’ x ‘Glacier’ population. The maps share three common markers and two other markers that have a common closest *B. napus* gene, and the cluster of markers that co-segregate with *Rlm2* span the *LepR3* locus (Figure 2).

### Candidate gene selection

Inspection of the 204 genes contained within the region of *B. rapa* A10 collinear to the *Rlm2* map interval lead to the identification of seven candidate gene homologues, selected on the basis of their potential roles in resistance to microbial pathogens. Two of the candidates (Bra008836, Bra008851) are homologous to *A. thaliana* genes involved in disease resistance signaling during infection by the bacterial pathogen *Pseudomonas syringae* [23]. The interval also contained Bra008930, previously identified as the *B. rapa* homologue of the *B. napus* blackleg resistance gene *LepR3* [11], and four other genes (Bra008869, Bra008870, Bra008910 & Bra008977) of unknown function in *A. thaliana* that are members of gene families involved in plant resistance responses (Table 1). Two of these, members of the Leucine Rich-Repeat (LRR) Protein family (Bra008869 & Bra008870) were examined using InterPro 5 and LRRfinder 2.0 and were both predicted to encode small (374 and 373 amino acids, respectively) proteins with predicted primary structures featuring signal peptides, LRR N-terminal domains, seven extracellular LRR domains and potential C-terminal LRR domains, similar to members of the plant defense-related polygalacturonase inhibitor protein (PGIP) family [24,25]. BLAST analysis showed these proteins shared 60% identity with PGIP-like family members from poplar (*Populus trichocarpa*).

**Table 1:** **Candidate defense-related** ***B. napus*** **genes identified within A10 region corresponding to the** ***Rlm2*** **map interval**

***B. napus*** **gene**	***A. thaliana*** **homologue**	***A. thaliana*** **description**	**Role**
BnaA10g19970D	At5g13320	PBS3 (avrPphB susceptible 3)	*P.syringae* defense response
BnaA10g20130D	At5g13160	PBS1 (avrPphB susceptible 1)	*P.syringae* defense response
BnaA10g30070D*	At5g12940	LRR Family Protein	Suppression of polygalacturonase?
BnaA10g30080D*	At5g12940	LRR Family Protein	Suppression of polygalacturonase?
BnaA10g20490D	At5g12180	CPK17 (Calcium-dependant protein kinase 17)	Unknown
BnaA10g20720D (*lepR3*)	At3g05650	AtRLP32 (Receptor-like Protein 32)	Homologue of *B. napus* gene *LepR3*
BnaA10g21210D	At5g11250	Disease Resistance Protein (TIR-NBS-LRR)	Unknown

Genes denoted with “*” are not anchored to *B. napus* A10 yet correspond to syntenic *B. rapa* A10 homologues.

Alignment of the *Rlm2* map interval to the *B. napus* after the release of the ‘Darmor-*bzh*’ reference genome [17] revealed a physical interval of approximately 873 kb spanning 191 genes (BnaA10g19800 – BnaA10g21710) containing syntenic homologues of 5 of the 7 candidate genes previously identified in *B. rapa*. The two ‘missing’ *B. rapa* homologues (PGIP-like genes Bra008869 & Bra008870) had been assigned to an unanchored “chrA10_random” pseudo-molecule, with the collinear *B. napus* A10 region spanning the genes in *B. rapa* being represented by a gap of approximately 43 kb (Figure 2, Table 1).

## Discussion

We were able to define the *Rlm2* locus to a map interval of 5.8 cM and a physical interval of 191 predicted *B. napus* genes, though further dissection of the *Rlm2* interval was hampered by a lack of polymorphism, as many additional markers located within the region proved to be monomorphic in our population. We could make efforts to produce additional markers targeted to the interval, however the recent advent of high-density marker systems for *B. napus*, such as DArT [26], Illumina Infinium 6 K [27,28] or 60 K ([29], Isobel Parkin, AAFC Saskatoon, unpublished data]) SNP arrays, or genotyping-by-sequencing methods [30] makes the pursuit of further specific sequence-characterised markers impractical. While we have provided several markers tightly-linked to the *Rlm2* locus which could be of use in blackleg resistance breeding programs, the real value of the work is in defining the physical location of *Rlm2* on chromosome A10. With this information any sequence-characterised marker set, including the high-density systems, can be orientated to the map in order to identify markers linked to the physical location of the gene.

The co-localisation of the blackleg resistance genes *LepR3* and *Rlm2* in the *B. napus* genome reported here draws an interesting comparison to the clustering of their corresponding avirulence genes, *AvrLm1* and *AvrLm2*, in the *L. maculans* genome [13]. Molecular characterisation of *Rlm2* and *AvrLm2* and comparison to the previously-identified *LepR3* [11] and *AvrLm1* [31] may provide some insight into the evolution of the molecular ‘arms race’ between plant and pathogen. We identified seven potential resistance-related homologues in the regions of *B. rapa* and *B. napus* genomes corresponding to the map interval containing *Rlm2* (Table 1). One of these genes, BnaA10g20720D, corresponds to the *LepR3* blackleg-resistance locus and was targeted in this study by the SCAR marker Ind10-20. This marker co-segregated with the resistance phenotype, as did two other markers corresponding to neighbouring genes (Figure 2). This suggests that *Rlm2* is located close to this region of the chromosome however, based on our results, we cannot rule out other candidate genes within the wider map interval (BnaA10g19800 – BnaA10g21710). These include a member of the Calcium-dependant protein kinase (CPK or CDPK) family (BnaA10g20490D). CPKs have been shown to be positive regulators of race-specific pathogen defense in several plant species [32-35]. They act to facilitate HR during ETI-mediated defense responses after translocation from the cytosol to the nucleus, where they activate WRKY transcription factors [36]. Other candidates include a member of the TIR-NBS-LRR class of resistance genes (BnaA10g21210D), which are well established as initiators of plant resistance responses during both direct and indirect interactions with pathogen effectors [37-40], and two genes (BnaA10g20130D & BnaA10g19970D) homologous to *A. thaliana* genes PBS1 and PBS3, respectively, involved in the *P. syringae* resistance response [23]. PBS1 encodes a receptor-like cytoplasmic kinase targeted for cleavage by the *P. syringae* effector AvrPphB, which triggers the CC-NBS-LRR class resistance protein RPS5 [41,42]. The final two candidate genes, BnaA10g30070D and BnaA10g30080D, encode putative PGIP-like proteins. PGIPs are small LRR-containing proteins that are secreted from the host cell into the apoplastic fluid where they inhibit host cell wall degradation by binding to pathogen endopolygalacturonases during infection by fungi [25], nematodes [43] and other plant pathogens. Though BnaA10g30070D and BnaA10g30080D were not incorporated in the main A10 chromosome build of the initial *B. napus* genome release, their *B. rapa* (Bra008869 & Bra008870) and *A. thaliana* (both match At5g12940) homologues are located in regions syntenic to the *Rlm2* map interval in their respective genomes [18,44].

The genomic interval containing *Rlm2* in the *B. napus* variety ‘Samouraï’ [6] was also shown to harbour a QTL for *L. maculans* resistance during field trials in France [6,45] despite the absence of the corresponding avirulence gene *AvrLm2* in French isolates [19]. It was suggested that either *Rlm2* has a residual effect on *avrLm2 L. maculans* isolates, or that another genetic factor limiting growth of the pathogen was linked to the *Rlm2* locus [6]. A number of the candidate defense-related genes identified here as being linked to the *Rlm2* locus could also be of interest as candidates in non-race specific adult-plant resistance. In particular, the interaction of plant PGIPs with pathogen polygalacturonases inhibits the degradation of the host cell wall and can also lead to accumulation of non-specific defense responses such as lignification and the production of reactive oxygen species [46]. Fungal polygalacturonases have been shown to accumulate in *B. napus* stems during infection by *L. maculans* and may play an important role in the development of canker lesions [47] though expression of the *L. maculans* polygalacturonase-encoding *pg*1 gene was not detected during cotyledon and leave infection [48]. If the candidate genes we identified do indeed encode functional PGIP proteins then they could potentially play a role in the suppression of blackleg disease in the adult plant.

The detailed genetic map and physical location of the *Rlm2* gene presented here should aid in the marker-assisted breeding of the gene into modern *B. napus* varieties. While *Rlm2* is of little use in Europe, due to the absence of the matching *AvrLm2* avirulence gene in most European *L. maculans* populations [49,50], it is a valuable source of resistance to blackleg disease in Canada. A survey of western Canadian isolates showed that 100% of isolates collected between 1997 and 2000, and 93.9% of isolates collected between 2003 and 2005, harboured *AvrLm2*. This high frequency was unexpected, as *Rlm2* has been available to Canadian plant breeders for some time and a greater adaptation by the pathogen was expected [51] suggesting *Rlm2* has not yet been widely deployed in Canadian *B. napus* varieties.

While *Rlm2* is potentially valuable in developing blackleg resistance canola varieties in North America, breeders must be cautious in its deployment. The lack of *AvrLm2* in current European *L. maculans* populations stands in stark contrast to the effectiveness of *Rlm2* in controlling blackleg in Europe several decades ago. High selection pressure has resulted in a dynamic evolution of race structure within the pathogen populations of Europe, leading to the sequential loss of *Rlm2*, *Rlm4* and *Rlm1*-mediated resistance [19,51,52]. A similar situation could develop in North America; an earlier survey of *L. maculans* isolates in southern Ontario, a winter-type canola growing region geographically isolated from the prairies of western Canada, found no *AvrLm2* isolates [53]. Kutcher et al. (2010) noted that the only *avrLm2* isolates detected in their survey were collected in southern Manitoba in 2003–2005. Another survey also found *avrLm2* isolates both in Alberta, Manitoba and directly south across the Canada/US border in North Dakota [54] and a recent report suggests nearly all *L. maculans* in North Dakota would be virulent on *Rlm2* varieties [55]. Clearly relying on *Rlm2* as the single resistance source for a canola variety would be foolhardy. Proper stewardship of the gene would entail pyramiding the resistance by combining *Rlm2* with other effective *R*-genes and/or quantitative resistance genetics [56].

## Conclusions

We are presented with three scenarios as to the identity of *Rlm2*; 1) the co-localisation of *Rlm2* and *LepR3* is due to the genes being allelic variants of the same gene locus (BnaA10g20720D), 2) *Rlm2* corresponds to one of the other *B. napus* candidate defense-related homologues identified within the syntenic map interval, or 3) *Rlm2* is a gene specific only to certain varieties of *B. napus* and not represented in the *B. napus* ‘Darmor-*bzh*’ or *B. rapa* var. ‘Chiifu’ genome sequences. Investigation of the candidate genes identified in this study is currently underway. Regardless of the molecular identity of *Rlm2*, delimiting the physical region of the *B. napus* genome that harbours the gene provides the information required for the efficient marker-assisted selection of *Rlm2* in modern canola breeding programs.

## Methods

### Mapping population

For the mapping of *Rlm2*, a BC_1_F_1_ population segregating for the *Rlm2* phenotype was produced by first creating F_1_ plants via a cross between the susceptible *B. napus* variety ‘Topas’ and the resistant *B. napus* doubled-haploid line ‘Glacier DH24287’ (*Rlm2*, *Rlm3*). A single F_1_ seedling was vernalised (4°C) for 8 weeks to ensure flowering, then backcrossed to ‘Topas’ to produce BC_1_F_1_ seeds.

### Phenotypic analysis

Seedlings were germinated in 96-cell trays containing an artificial soil mix [57] in a controlled growth chamber (20°C, 16 h days, light intensity c. 450 μmol m^−2^ s^−1^ at bench level, and 18°C, 8 h nights). The cotyledons of 7 day-old seedlings were inoculated with a pycnidiospore suspension of *L. maculans* isolate ‘165’ (*avrLm1*, *AvrLm2*, *avrLm3*) from the Rimmer Collection, AAFC Saskatoon, which is virulent on ‘Topas’ (no effective blackleg resistance) and avirulent on ‘Glacier DH24287’ (*Rlm2*, *Rlm3*). A small wound was made in the centre of each cotyledon lobe and 10 μL of 2×10^7^ spores/mL suspension was applied to each wound (4 infection sites per seedling). The resistance phenotype of the seedlings was rated at 14 days post-infection using a 0–9 scale [58]; where ratings of 0–4 (induced HR) are classified as ‘resistant’, 5 as ‘intermediate’ and 6–9 (no HR) as ‘susceptible’. To confirm that the resistance screening of the BC_1_F_1_ population was detecting the *Rlm2* gene and not *Rlm3 or LepR3*, the “*avrLm1*, *Avrlm2*, *avrLm3*” pathotype of ‘165’ was tested using 4–8 seedlings each (8–16 cotyledons per test) of the additional *B. napus* control lines ‘Topas DH16516’ (a doubled-haploid line of ‘Topas’), ‘Tapidor DH’ (*Rlm2*), ‘Columbus’ (*Rlm1*, *Rlm3*), ‘Quantum’ (*Rlm3*) and the transgenic line ‘NLA8-2’ (*LepR3*).

### Marker selection

An initial group of 218 BC_1_F_1_ individuals were assessed for their reaction to the *L. maculans* isolate ‘165’. DNA was extracted using DNeasy 96 Plant Kits (QIAGEN Inc., USA) or as described by Larkan *et al*. (2013). Thirty BC_1_F_1_ individuals (15 resistant, 15 susceptible) were selected in order to screen microsatellite markers (http://aafc-aac.usask.ca/BrassicaMAST/) spanning chromosome A10, based on the previously described map location of *Rlm2* [6]. This identified markers that were both polymorphic in the population and linked to the *Rlm2* locus. Microsatellite marker reactions were performed as described by [59] and genotyping was performed using a MegaBACE capillary sequencer (GE Health, Canada).

### Fine mapping

Further screening was performed to expand the mapping population; in total of 940 BC_1_F_1_ seedlings were phenotyped for their reaction to ‘165’and screened for recombination between the *Rlm2*-flanking markers. Putative recombinant individuals were selected, vernalised and allowed to set selfed seed (BC_1_F_2_).

To confirm the *Rlm2* phenotype and marker genotypes for the recombinant BC_1_F_1_ selections, between 8 and 12 BC_1_F_2_ seedlings per line were infected with ‘165’, and DNA produced from bulked BC_1_F_2_ tissue was used to confirm the genotypes for each marker. To enrich the map, an additional SCAR marker (“Ind10-20”), designed and run as previously described [11], was targeted to the *LepR3* locus (Bra008890) using the following primers; F - TTCGTGATGAGTTTGCGGTTC, R -CAGTCGCTGTTATTCACCCATGA. Additionally, a single CAPS marker, “CAPS94”, was designed and added to the map, taking advantage of a differential restriction site for the enzyme BstUI in the Glacier DH24287 homologue of Bra008928. CAPS alleles were PCR amplified (95°C, 5:00; (95°C, 0:30; 55°C, 0:30; 72°C, 1:00) x 35 cycles; 72°C, 10:00; F primer – CCTTCCTGGGGGAAAACTAA, R primer - TCGTAGCGTTTCCTCCAAAC) using AmpliTaq Gold Master Mix (Life Technologies, USA) before digestion with BstUI restriction enzyme (New England Biolabs, USA). Digested CAPS products were resolved on 1.5% agarose gels.

A final linkage map of the *Rlm2* interval was constructed after placing markers in order based on their homologous *B. rapa* position and integrating the *Rlm2* phenotypic data. Map distances were calculated manually (cM_BC_ = (x/n) *100, where x = recombination events and n = total population size) and parsimonious marker order was confirmed using the MAP function of QTL IciMapping v3.2 software [60].

### Comparison to *LepR3* map and identification of *Rlm2* candidate homologues in *B. rapa* and *B. napus*

During the mapping process, performed concurrently with the mapping of *LepR3* [11], it became apparent that both genes mapped to a similar region of chromosome A10. Additional markers from the *LepR3* map were tested for polymorphism in the ‘Topas’ x ‘Glacier’ population and integrated into the *Rlm2* map. Markers common to both studies were used to align the maps and assess the relative positions of the genes.

For each marker placed on the map, the sequence used to create the marker was initially matched to its homologous region of the *B. rapa* genome [18] using BLASTN [61] in the BRAD *Brassica* Database portal [62] and the matching or closest *B. rapa* gene recorded. Homologous genes occurring within the *Rlm2* map interval were examined and pathogen defense-related genes or gene family members were considered as candidates. Marker positions and candidate genes were later reassessed in relation to the recently-released *B. napus* genome [17]. Additional annotation of candidate proteins was performed using the online tools InterProScan 5 [63] and LRRfinder 2.0 [64].

## Abbreviations

CAPS: Cleaved amplified polymorphic sequence
LRR: Leucine-rich repeat
NBS: Nucleotide binding site
PCR: Polymerase chain reaction
PGIP: Polygalacturonase inhibitor protein
SCAR: Sequence characterised amplified region
